# Preparation and Characterization of Protocatechuic Acid Sulfates

**DOI:** 10.3390/molecules24020307

**Published:** 2019-01-16

**Authors:** Sofia M. Gutierrez-Zetina, Susana Gonzalez-Manzano, Jose J. Perez-Alonso, Ana M. Gonzalez-Paramas, Celestino Santos-Buelga

**Affiliations:** Grupo de Investigación en Polifenoles, Unidad de Nutrición y Bromatología, Facultad de Farmacia, Universidad de Salamanca, Campus Miguel de Unamuno, 37007 Salamanca, Spain; sofia_martinez@usal.es (S.M.G.-Z.); susanagm@usal.es (S.G.-M.); josejpa@usal.es (J.J.P.-A.); paramas@usal.es (A.M.G.-P.)

**Keywords:** polyphenols, phenolic metabolites, phase II conjugates, phenolic acids, sulfates, hemisynthesis, identification

## Abstract

Protocatechuic acid (3,4-dihydroxybenzoic acid; PCA) is a phenolic acid present in plants as a secondary metabolite and is also produced in the human organism as a metabolite from the degradation of polyphenols by the intestinal microbiota, particularly of flavonoids. However, PCA, like most polyphenols, is biotransformed in the human body to different conjugates as sulfates, which are found circulating in blood and could be involved in the bioactivity of the original compound. This paper describes a simple process for the preparation of PCA monosulfates with satisfactory yields. Two compounds were obtained that were identified as PCA-3-sulfate and PCA-4-sulfate by mass spectrometry and ^1^H and ^13^C nuclear magnetic resonance using one- and two-dimensional techniques (heteronuclear single-quantum coherence and heteronuclear multiple-bond correlation). Differential MS fragmentation behavior and UV spectra were observed for each compound, which could be used for their identification in samples of unknown composition. The described procedure can be used for the preparation of these polyphenol metabolites in view of their use in in vivo and in vitro studies, as well as standards for their analysis in biological fluids, to contribute to the elucidation of biological effects of dietary polyphenols.

## 1. Introduction

Most polyphenols are poorly absorbed and largely biotransformed in the human body, either by phase I and II enzymes of the metabolism or by gut microbiota, so that metabolites represent the main circulating forms of dietary polyphenols in the organism and may ultimately be responsible for the reported bioactivity of the original compounds [1]. Most of the consumed polyphenols usually reach the gut, where they interact with the microbiota and can be degraded to a range of products that may be bioactive [2,3,4]. As a matter of fact, the concentrations in plasma and urine of microbial phenolic metabolites are normally higher than those of the metabolites derived from polyphenols uptake in the stomach or small intestine [5,6,7,8,9]. In general, phenolic sulfates have been indicated as abundant metabolites of polyphenols in human plasma [10].

Protocatechuic acid (3,4-dihydroxybenzoic acid; PCA) is a phenolic acid present in plants as a secondary metabolite produced through the shikimic acid pathway [11]. As such, it is commonly found in fruits, vegetables, grains and spices, such as grapes, berries, onions, brown rice, rosemary, and cinnamon, among others [12]. PCA is also produced in the human organism as a metabolite from the degradation of polyphenols by the intestinal microbiota, particularly of flavonoids like anthocyanins, flavonols or procyanidins [13]. It may be absorbed through the intestinal epithelium and reach systemic circulation. After uptake, PCA undergoes structural modifications through conjugation processes, mostly occurring in the liver, giving rise to sulfated and glucuronidated forms that can be distributed to the tissues, recycled back to the intestine, or excreted in urine [14,15]. PCA conjugates have been detected in relevant concentrations in plasma, urine, and feces after consumption of polyphenol-rich diets [7,8,9,10,16]. Concentrations of phase II conjugates (sulfates + glucuronides) of PCA up to 5,540 ± 490 nM were determined in urine by Czank et al. [7] following consumption of 500 mg of ^13^C-labelled cyanidin-3,*O*-glucoside by healthy humans, reaching maximum level 24 h after intake. In the same intervention study, maximum concentrations of PCA sulfates of 157 ± 116 nM were found in serum with a t_max_ of 11.4 ± 3.8 h, while maximum urinary concentrations of 1112 ± 318 nM and 1244 ± 333 nM were found for PCA-3-sulfate and PCA-4-sulfate, respectively, 1–2 h after anthocyanin intake [9]. Both sulfates were also detected in feces with maximum recoveries of 30.0 ± 27.7 µg (PCA-3-sulfate) and 23.0 ± 18.1 µg (PCA-4-sulfate) at 6–24 h post-bolus from 500 mg of anthocyanidin consumption [9]. De Ferrars et al. [8] found that PCA sulfates constituted the main group of phenolic metabolites in plasma, representing 28.31% of total detected metabolites 3 h after an acute intake of 500 mg of an elderberry extract by post-menopausal women. Concentrations of PCA sulfates determined at 3 h after intake were 2014 ± 1765 nM/mM creatinine and 358 nM in urine and plasma, respectively, higher than those of PCA glucuronides (495 ± 190 nM/mM creatinine and 29 nM) and non-conjugated PCA (1534 ± 1232 nM/mM creatinine and 24 nM) [8].

Different biological and pharmacological activities have been ascribed to PCA, including antioxidant, antimicrobial, anticancer, antiulcer, antidiabetic, antiaging, anti-inflammatory, and analgesic properties, as well as cardiovascular, hepatic, neurological, and nephron protective effects [11,12]. As for PCA sulfates, some authors have reported anti-inflammatory effects in in vitro assays. Thus, PCA sulfates have been shown to reduce the production of proinflammatory biomarkers of coronary risk, such as interleukin-6 (IL-6) [17] and soluble vascular cell adhesion molecule-1 (VCAM-1) in human endothelial cells [17,18]. In addition, PCA-3-sulfate was demonstrated to significantly reduce liposaccharide induced tumor necrosis factor alpha (LPS-TNF-α) secretion and decrease interleukin-1 beta (IL-1β) in THP-1 monocytes [19].

A common problem when studying the effects and activity of conjugated metabolites of phenolic compounds, such as PCA sulfates, is the usual lack of commercial standards, so they must be prepared in the laboratory. Procedures for the chemical hemisynthesis of sulfates of different hydroxybenzoates that are common metabolites of flavonoids, including protocatechuic acid, were previously described by Zhang et al. [20] and Almeida et al. [21]. The method by Zhang et al. [20] involved the selective protection of hydroxyl groups in the form of benzyl esters or *tert*-butyldimethylsilyl ethers and further sulfation with trichloroethyl (TCE) chlorosulfate; finally, the protecting groups (TCE, benzyl, silyl) were removed by hydrogenation under mild reducing conditions and in HF-pyridine in THF to yield the corresponding sulfate derivatives. Sulfate derivatives were synthesized by Almeida et al. [21] by reaction of phenolic precursors with sulfur trioxide-pyridine in anhydrous pyridine and further purified on a Dowex 50W-X8 ion-exchange resin. The products were then converted into their sodium salts to improve their stability.

In the present study, another feasible and easily reproducible method using sulfur trioxide-*N*-triethylamine as sulfation reagent was proposed for the preparation of PCA sulfates. Furthermore, UV absorption and mass spectral characteristics, NMR data for their structural characterization, as well as information about their stability are provided. This knowledge is expected to contribute to the correct identification of these metabolites in biological samples. Furthermore, the proposed procedure can be used for the preparation of PCA sulfates in view of their employment in studies on their biological activity and mechanisms of action.

## 2. Results and Discussion

### 2.1. Preparation of PCA Sulfates

PCA sulfates were prepared via the reaction of the precursor phenolic acid with sulfur trioxide-*N*-triethylamine, based on a method previously described for the preparation of flavonoid sulfates [22]. The reaction was kept for 3 h, at which it was verified that the formation of the sulfates reached their maximum, as checked by HPLC. The obtained chromatograms showed a not-well resolved mixture of peaks presenting pseudomolecular ions [M − H]^−^ at *m*/*z* 313 and 233, coherent with PCA disulfates and monosulfates, respectively, together with some remaining PCA ([M − H]^−^ at *m*/*z* 153).

**Figure 1:**
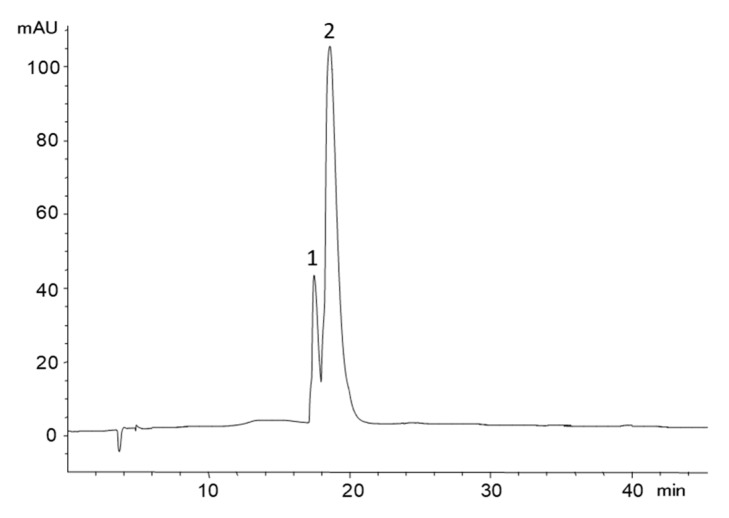
HPLC chromatogram recorded at 250 nm corresponding to the mixture of protocatechuic acid (PCA) monosulfates obtained after semipreparative HPLC fractionation. Peak identification: (1) PCA-4-sulfate, and (2) PCA-3-sulfate.

Semipreparative HPLC of this crude mixture allowed obtaining different fractions with a mixture of the two monosulfates in different proportions, but none of them contained a single compound. The close elution and difficult chromatographic separation of PCA-3-sulfate and PCA-4-sulfate have also been noticed by other authors [8,10,16,21]. Fractions containing the monosulfates were pooled to a unique fraction, which was concentrated and passed through an ion exchange cartridge, Oasis MCX, to remove residual trimethylamine; the sulfates were further converted into their sodium salts and freeze-dried. Finally, 128 mg of a clean fraction containing the two PCA monosulfates was obtained as sodium salts, which represented a final yield of 15.5%. Figure 1 shows the HPLC chromatogram of the obtained extract.

Compared to previously described procedures for the synthesis of benzoic acid sulfates [20,21], the method proposed herein is faster and simpler, and it only involves one reaction step. A different sulfation reagent (i.e., sulfur trioxide-*N*-triethylamine) is employed and the use of pyridine is avoided, making it safer and facilitating the removal of the solvent and reagent excess. In preliminary assays carried out to optimize the method, it was also checked that partial losses of the products were produced during the elimination of pyridine, when it was used as a solvent, as also reported by Almeida et al. [21].

### 2.2. Absorption and Mass Spectral Characteristics

**Figure 2:**
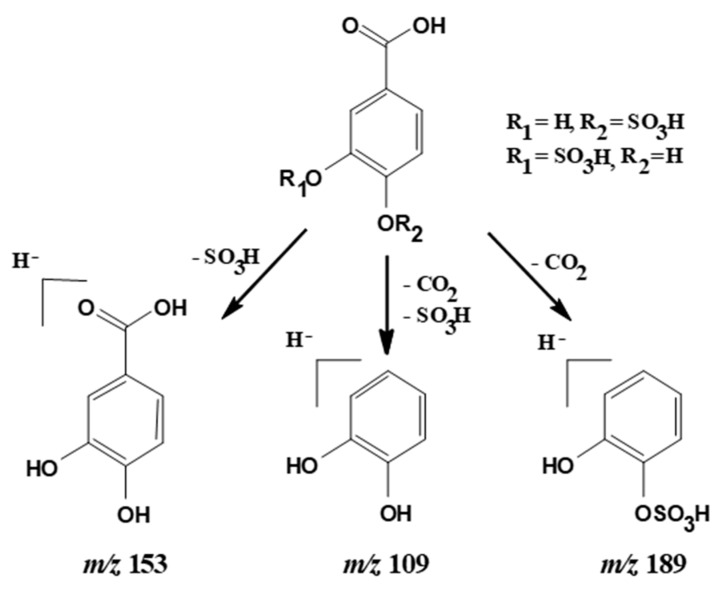
MS^2^ fragmentation of the PCA monosulfates (pseudomolecular ion [M − H]^−^ at *m*/*z* 233).

**Figure 3:**
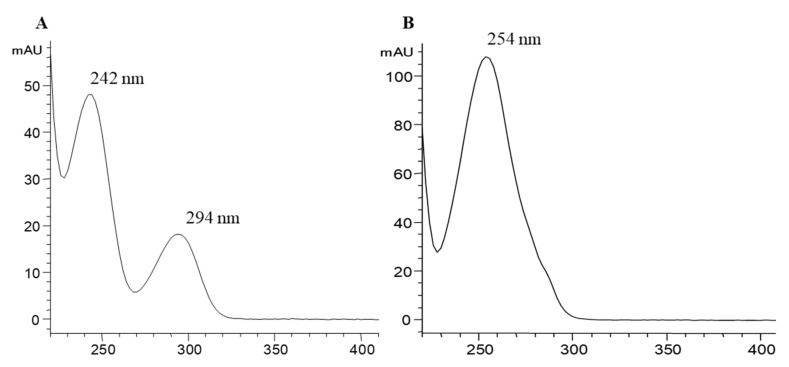
UV spectra of peaks 1 (**A**) and 2 (**B**) in the chromatogram of Figure 1.

ESI/MS analysis of peaks 1 and 2 confirmed that they had the same pseudomolecular ion [M − H]^−^ at *m*/*z* 233, corresponding to PCA monosulfates, and also showed the same MS^2^ fragmentation pattern yielding product ions at *m*/*z* 189, 153, and 109. The ion at *m*/*z* 189 can be attributed to the loss of CO_2_ ([M − H − 44]^−^) from the carboxyl group of the PCA, indicating that this functional group was not substituted and allowing the confirmation of the phenolic hydroxyl groups as location for the sulfate moieties. The fragment at *m*/*z* 109 was due to the further loss of the sulfate group ([M − H − 44 − 80]^−^) (Figure 2). For both compounds, the main MS^2^ fragment was at *m*/*z* 153 (PCA) from the loss of the sulfate group ([M − H − 80]^−^), but there were some differences in the relative abundance among the distribution of the other two fragment ions. For peak 1, the abundance of ion at *m*/*z* 109 was 74%, and at *m*/*z* 189 was 5%, while for peak 2 the abundance of ion at *m*/*z* 109 was 78% and at *m*/*z* 189 was 4%. Interestingly, the two peaks also possessed different UV spectra (Figure 3). Peak 1 presented two bands of maximum absorption at 242 nm and 294 nm, whereas peak 2 showed λmax at 254 nm. The existence of different fragmentation behavior and spectral shapes are characteristic features that might allow the assignment of peak identities in a given sample with unknown composition.

### 2.3. NMR Analysis

The NMR spectrum of the PCA monosulfates mixture showed two series of analogous peaks, with two doublets and one singlet for each series. The COSY, HMQC and HMBC plots (see Figures S1–S3, included as supplementary information) did not show interactions between the two series of peaks, which confirmed the existence of two different compounds. ^1^H and ^13^C-NMR data and assignments of the displacements of the protons and carbons in each position of the aromatic ring, made based on the two-dimensional spectra ^1^H-^13^C HMBC and HSQC, are shown in Table 1. The obtained results agreed with data predicted by the Perkin Elmer ChemDraw Professional 16.0.1.4 (77) software, with minor modifications in the displacement of the protons in position 2 for PCA-3-sulfate and in position 5 for PCA-4 sulfate. This difference was explained by the influence of the adjacent sulfate group, which has free rotation and can adopt a conformation in which the interaction is greater than theoretically expected. The major compound was assigned to PCA-3-sulfate, for which it was found that the proton in position 2 appeared at 7.76 ppm, undergoing a more significant shift to a lower field than would theoretically be expected (6.85 ppm), while the proton H5 did not change over the expected displacement. In the minority compound, PCA-4-sulfate, proton H5 underwent a lower field displacement (7.14 ppm) than theoretically expected (6.85 ppm), which could be explained by the interaction of the adjacent sulfate group. The two sulfates showed a proportion of 1:2.5, as determined by NMR. The identification of PCA-3-sulfate as the major compound allowed it to be assigned to the peak 2, which showed the largest area in the chromatogram of Figure 1; consequently, PCA-4-sulfate, the compound with lower NMR signal, was assigned to peak 1.

### 2.4. Stability of the Freeze-Dried Sulfates

The prepared compounds were freeze-dried and stored in a desiccator at room temperature for three months. On days 1, 15, 30, 60, and 90, 2 mg of the preparation was weighed and dissolved in ultrapure water to obtain a final concentration of 0.1 mg/mL and analyzed by HPLC to check stability. According to the peak areas recorded at 250 nm (Table 2), it can be considered that the freeze-dried compounds only kept reasonable stability for around 15 days (88% of the initial peak area for PCA-4-sulfate and 85% for PCA-3-sulfate) to then fell quickly, losing up to 98% of their initial concentration at day 30 of storage. This loss was mainly attributed to the cleavage of the sulfate moiety to release PCA, as observed in the chromatograms. The stability of both compounds was very similar between them.

## 3. Materials and Methods

### 3.1. Standards and Reagents

Protocatechuic acid (PCA), dioxane, trifluoroacetic acid (TFA), acetonitrile, and sulfur trioxide-*N*-triethylamine were purchased from Sigma-Aldrich (Darmstadt, Germany). Methanol was purchased from Macron Fine Chemicals^TM^ (Gliwice, Poland), formic acid and ammonia from VWR (Fontenay-sous-Bois, France), and sodium hydroxide from Panreac (Barcelona, Spain).

### 3.2. Preparation of PCA Sulfates

The procedure was based on the method previously described in our laboratory for the hemisynthesis of catechin sulfates [22] with modifications. PCA (500 mg) was dissolved in dioxane (50 mL) and allowed to react with a 10-fold molar excess of sulfur trioxide-*N*-triethylamine. The reaction was carried out under an argon atmosphere in a water bath (40 °C) for 3 h. The sulfation products precipitated out and the supernatant was decanted. The precipitate was re-dissolved in ultrapure water and dried on a rotary evaporator. The composition of the extract containing a sulfate mixture was analyzed by HPLC-DAD-MS, as described in Section 3.3. Products of sulfation were newly recovered in a minimal volume of ultrapure water, and PCA monosulfates were further fractionated by semi-preparative HPLC, as described below. The fractions containing the monosulfates were collected, concentrated, and re-dissolved in 2% formic acid in ultrapure water and loaded into an ion exchange cartridge, Oasis MCX 3 cc (60 mg), to remove residual trimethylamine. The cartridges were conditioned with methanol and equilibrated with ultrapure water; the sample was loaded and eluted with 2% formic acid in ultrapure water, and the sulfates were collected in this step. For greater stability, the sulfates were converted into their sodium salts as described by Almeida et al. [21], with 0.5 M sodium hydroxide, and freeze-dried. The identity and purity of the compounds were checked by NMR, as described below. The stability of the freeze-dried compounds was evaluated by HPLC from the areas of their chromatographic peaks recorded at 250 nm for 3 months.

### 3.3. HPLC-DAD-MS Analyses

An Agilent 1200 series HPLC system (Agilent Technologies, Palo Alto, CA, USA) provided with a quaternary pump and a diode array detector (DAD) and controlled by the ChemStation software (version B.04.01) was used. The column was an Agilent Poroshell 120 EC-C18, 2.7 μm (4.6 mm × 150 mm) thermostatted at 35 °C. Solvents were (A) 0.1% formic acid in ultrapure water, and (B) acetonitrile, establishing the following elution gradient: 100% A in 10 min, 0–5% B in 10 min, 5–10% B in 15 min, 10–14.5% B in 20 min, and 14.5–60% B in 15 min, at a flow rate of 0.5 mL/min. Double detection was carried out in the diode array spectrophotometer and by mass spectrometry (MS), using 250 and 290 nm as preferred wavelengths for chromatogram recording. MS detection was performed on an API 3200 Qtrap (Applied Biosystems, Darmstadt, Germany) equipped with an electrospray ionization probe (ESI) and a triple quadrupole mass analyzer that could also act as an ion trap, controlled by Analyst 5.1 software. Mass spectra were recorded in negative ion mode between *m*/*z* 100 and 900. Zero air at 50 psi was used as a nebulizer, turbo gas at 500 °C and 40 psi for the elimination of the eluent, and nitrogen at 25 psi as a curtain and medium-collision gas. The method of full scan at high sensitivity (Enhanced MS, EMS) was used for data acquisition, followed by an analysis in Enhanced Product Ion (EPI) mode to obtain the characteristic fragmentation of the majority ion obtained in the first experiment. The EMS parameters were as follows: capillary voltage, −3500 V; de-clustering potential (DP), −65 V; entrance potential (EP), −10 V; and collision energy (CE), −20 V. The conditions in the EPI mode were: DP, −40 V; EP, −8 V; CE, −50 V; and collision energy spread (CES), 20 V.

### 3.4. Semipreparative HPLC

The separation was performed with an Agilent 1260 Infinity LC equipment (Agilent Technologies, Waldbronn, Germany), consisting of a thermostatted autosampler, a binary system with two coupled preparative pumps, a diode array detector, and a collector thermostatic sampling, controlled by the OpenLab CDS Chemstation Workstation software (version C.01.04). An Agilent Prep-C18 column, 5 μm (21.2 mm × 150 mm) was used. The solvents were (A) 0.05% trifluoroacetic acid in ultrapure water, and (B) acetonitrile. The elution gradient was 100% A for 30 min, 0–5% B 10 min, 5–10% B for 20 min, 10–60% B for 5 min, using a flow rate of 15 mL/min. Chromatograms were acquired at 290 nm for peak collection.

### 3.5. NMR Analysis

The ^1^H NMR (400 MHz) and ^13^C-NMR (100 MHz) spectra of the isolated metabolites were measured in deuterated DMSO on a Bruker Avance DRX-400 spectrometer (Bruker Biospin GmbH, Rheinstetten, Germany) at 298 K. The resonances at 2.50 ppm of the residual in the ^1^H spectra and at 39.79 ppm for deuterated DMSO in the ^13^C spectra were used as internal references. ^1^H chemical shifts were assigned using one-dimensional (1D) and two-dimensional (2D) ^1^H NMR (correlation spectroscopy (COSY)), while ^13^C resonances were assigned using 2D NMR (heteronuclear multiple-bond correlation (HMBC) and heteronuclear multiple-quantum coherence (HMQC)). COSY, HMQC and HMBC plots are included as supplementary information in Figures S1–S3, respectively.

## 4. Conclusions

Protocatechuic acid monosulfates were synthesized, isolated, and characterized by HPLC-DAD-ESI/MS and NMR. Two compounds were obtained that were identified as PCA-4-sulfate and PCA-3-sulfate, respectively, based on their NMR and mass spectral characteristics. Compared to previously described procedures, the hemisynthesis method proposed herein is simpler, involving only a reaction step. The sulfation reagent (sulfur trioxide-*N*-triethylamine) and solvent used are easier to remove and safer, and the reaction kinetics is faster. The prepared metabolites are of great interest for their use in in vivo and in vitro studies, to contribute to the knowledge of the compounds and mechanisms involved in the biological effects of dietary polyphenols. The prepared PCA-sulfates and data that contributed to their analytical features, such as their differential MS fragmentation behavior and UV spectra, are also helpful as analytical standards for the identification of phenolic metabolites in samples of unknown composition.

## Figures and Tables

**Table 1:** ^1^H and ^13^C-NMR data and HMBC correlations obtained for PCA-3-sulfate, and PCA-4 sulfate determined in deuterated DMSO.

Position.	δ ^1^H (ppm); m; *J* (Hz)	δ ^13^C (ppm)	HMBC
**PCA-3-Sulfate**			
1		125.2	H2, H5
2	7.76, s *	124.1	H6
3		140	H2, H5, H6
4		152.3	H2, H5, H6
5	6.80, d, *J* = 8.0	116.2	H6
6	7.50, d, *J* = 8.4	126.5	H2, H5
Carbonyl		169	H2, H6
**PCA-4-Sulfate**			
1		148.1	H2, H5
2	7.40, s	118.1	H6
3		148.1	H5, H2
4		143.2	H2, H6, H5
5	7.14, d, *J* = 9.2	121.4	
6	7.31, d, *J* = 9.8	120.7	H2
Carbonyl		169	H2, H6

s *, singlet; d, doublet.

**Table 2:** Evolution of the areas (mAU) of the chromatographic peaks of PCA-4-sulfate and PCA-3-sulfate recorded at 250 nm over 3 months of storage of the freeze-dried compound at room temperature.

Time (days)	PCA-4-Sulfate (mAU)	PCA-3-Sulfate (mAU)
1	1203.6 (100% *)	5940.4 (100%)
15	1062.3 (88%)	5095.5 (85%)
30	23.4 (2%)	163.8 (2.7%)
60	18 (1.5%)	123.6 (2%)
90	0 (0%)	132.3 (2.2%)

* in brackets it is indicated the percentage of peak area relative to day 1.

## Supplementary Materials

Supplementary NMR data are included in Figures S1–S3.
