# Supplementary material for: Preparation and Characterization of Protocatechuic Acid Sulfates
Source: Molecules. 2019 Jan 16;24(2):307. doi: 10.3390/molecules24020307 (PMC6359441; doi:10.3390/molecules24020307)
Supplement: Supplementary file 1 [file molecules-24-00307-s001.zip › Supplementary files/Figure_S1.pdf]

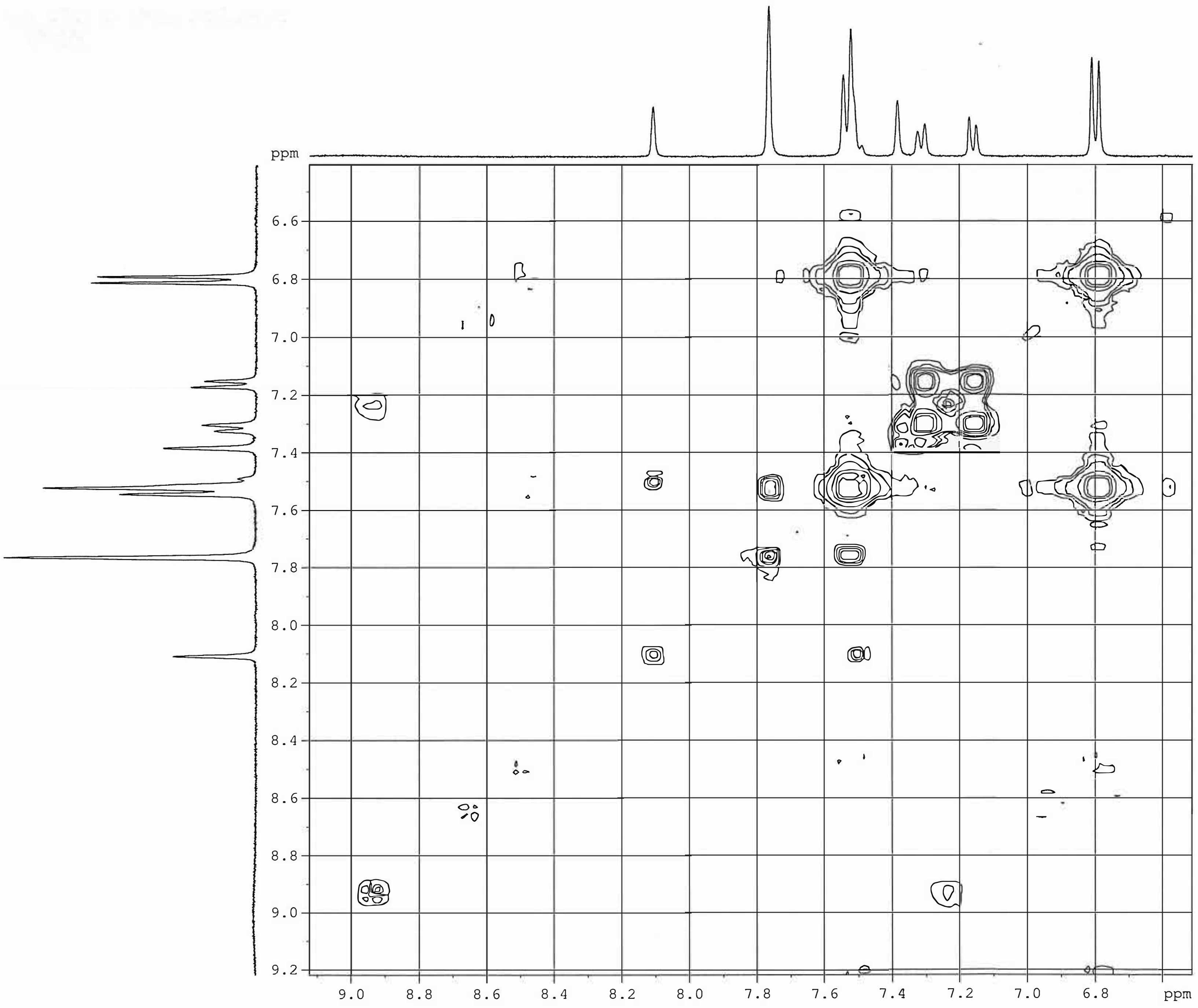

Figure S1. ^{1}H- ^{1}H COSY NMR correlation spectroscopy 2D NMR spectrum of PCA-3-sulfate, and PCA-4 sulfate (400 MHz, DMSO-d6).
